# Supplementary material for: OncoScape: Exploring the cancer aberration landscape by genomic data fusion
Source: Sci Rep. 2016 Jun 20;6:28103. doi: 10.1038/srep28103 (PMC4913322; doi:10.1038/srep28103)
Supplement: Supplementary Information [file srep28103-s1.doc]

**Supplementary Information**

­­OncoScape: Exploring the cancer aberration landscape by genomic data fusion

Andreas Schlicker^1^, Magali Michaut^1^, Rubayte Rahman^2^ and Lodewyk FA Wessels^1,3,*^

^1^Division of Molecular Carcinogenesis, Netherlands Cancer Institute, Amsterdam, The Netherlands
^2^Research IT, Netherlands Cancer Institute, Amsterdam, The Netherlands
^3^Faculty of EEMCS, Delft University of Technology, Delft, The Netherlands

^*^Correspondence to: Lodewyk FA Wessels, Plesmanlaan 121, 1066 CX Amsterdam, The Netherlands, phone: +31 20 512 7987, email: [l.wessels@nki.nl](mailto:l.wessels@nki.nl)

**The global aberration landscape of tumors**

Most genes were found to be aberrated in at least one data type in at least one cancer type (Table S1). However, these numbers dropped quickly when focusing on genes that showed aberrations in several data types. Only 537 or 659 genes had an oncogene or tumor suppressor score, respectively, of three in at least one cancer type, meaning aberrations in at least three data types. The number of genes with a certain score was comparable across most cancer types (Table S1), with the most notable exceptions being rectal cancer (READ) and head and neck squamous cell carcinoma (HNSC). For READ, the small dataset likely contributed to this apparently low number of called aberrations (Table 1). However, the size of the dataset available for HNSC was comparable to other cancer types. A score of four was the highest reached by any gene for oncogene and tumor suppressor score. The well-known tumor suppressor genes *RB1* received the highest score as tumor suppressor for breast (BRCA), ovarian cancer (OV) as well as lung adenocarcinoma (LUAD) (Table S2). *BRCA2* and *PTEN*, also established tumor suppressor genes, were scored highly as tumor suppressors in both BRCA and GBM. One of the genes with the highest oncogene score in KIRC was *TRRAP*, which has been suggested as an essential cofactor for the c-MYC and E1A/E2F oncogenic transcription factor pathways^1^.

Several chromosomes exhibited characteristically high tumor suppressor scores or oncogene scores. Interestingly, individual genes on these chromosomes were aberrated in the opposite direction. For example, Chromosome 9 showed mostly inactivating aberrations while the oncogene scoring of CA9, the gene with the highest oncogene score on this chromosome, is in line with the published literature^2–4^. A similar phenomenon could be observed for Chromosome 20. Most genes on this chromosome had activating aberrations while *SSTR4*, located on 20p, showed the highest tumor suppressor score on Chromosome 20. Previous reports have shown that activation of *SSTR4* leads to arrest in the G_1_-phase of cell cycle^5^, which is in line with the classification as tumor suppressor gene in our analysis.

**Pathway aberration patterns**

We identified several pathways that were targeted by similar aberrations across all cancer types, for instance calcium signaling (Figure 4a). The types of aberrations found in genes belonging to NF-Kappa B signaling, on the other hand, were strongly dependent on the cancer type. The detailed aberration profile clearly showed the tissue dependence of the type of aberrations for most genes (Figure S9). Overall, this led to a strong oncogenic signal in GBM and strong tumor suppressor signals in bladder urothelial carcinoma (BLCA) and BRCA. Some known oncogenic pathways, such as PI3K-Akt signaling, showed overall slightly higher tumor suppressor than oncogene scores. This could be an effect caused by different branches of the pathway. The signaling axis from growth factor receptors through PI3K and AKT towards cell cycle exhibited a strong oncogenic signal in almost all cancer types (Figure S10). Other branches of the pathway, however, were much more heterogeneous leading to the observed overall scoring.

When comparing the aberration patterns observed in tumor samples and cell lines, we observed highly correlated aberrations in some tissues, such as GBM, BRCA and COAD. Looking at individual pathways, the NF-Kappa B signaling pathway in COAD illustrates this very well (Figure S12). The large majority of genes in this pathway were scored highly similarly in tumor samples and cell lines and only few genes switched from oncogene to tumor suppressor or vice versa. This clearly shows that available colon cancer cell lines recapitulate changes found in primary tumor very closely. For other cancer types, pathway aberration patterns in tumor samples and cell lines showed little to no correspondence. In HNSC, VEGF signaling was overall scored as an oncogenic pathway in the tumor samples but scored as a tumor suppressor pathway in cell lines. Although, p38 MAP kinases were the only genes with contradictory aberration patterns in HNSC tumors and cell lines on the VEGF signaling pathway, any aberrations in roughly half of the genes were unique to either tumor samples or in cell lines (Figure S13). These genes were predominantly targeted by activating aberrations in tumors and inactivating aberrations in cell lines resulting in the apparent overall difference.

Table S1: Number of genes with scores above different thresholds. In case of the combined score (calculated as oncogene score (OG) – tumor suppressor gene score (TS)), the absolute value was taken into account.

|  | score >= 1 | | | score >= 2 | | | score >= 3 | | | score >= 4 | | |
| --- | --- | --- | --- | --- | --- | --- | --- | --- | --- | --- | --- | --- |
|  | Combined  score | OG  score | TS  score | Combined score | OG  score | TS  score | Combined  score | OG  score | TS  score | Combined  score | OG  score | TS  score |
| Any cancer | 20919 | 18391 | 19459 | 6767 | 5946 | 706 | 483 | 537 | 659 | 8 | 8 | 12 |
| BLCA | 8629 | 6033 | 7378 | 777 | 683 | 744 | 17 | 21 | 20 | 0 | 0 | 0 |
| BRCA | 10178 | 10108 | 11673 | 1445 | 2112 | 2484 | 91 | 156 | 209 | 2 | 0 | 4 |
| COAD | 9431 | 6005 | 7478 | 1491 | 896 | 1444 | 103 | 69 | 114 | 4 | 5 | 0 |
| GBM | 12001 | 8014 | 8272 | 1869 | 1429 | 1283 | 112 | 115 | 70 | 0 | 0 | 1 |
| HNSC | 6626 | 6591 | 8307 | 302 | 463 | 629 | 2 | 1 | 5 | 0 | 0 | 0 |
| KIRC | 9412 | 7336 | 9417 | 947 | 1107 | 1526 | 48 | 75 | 111 | 2 | 3 | 6 |
| LUAD | 9464 | 8085 | 9551 | 948 | 1202 | 1428 | 43 | 58 | 93 | 0 | 0 | 2 |
| LUSC | 9482 | 7409 | 7621 | 1094 | 1221 | 1090 | 39 | 54 | 48 | 0 | 0 | 0 |
| OV | 9220 | 6474 | 6055 | 846 | 844 | 717 | 21 | 36 | 20 | 0 | 0 | 1 |
| READ | 2342 | 900 | 1475 | 7 | 7 | 3 | 0 | 0 | 0 | 0 | 0 | 0 |
| UCEC | 7324 | 6867 | 7748 | 623 | 725 | 886 | 33 | 29 | 47 | 0 | 0 | 0 |

Table S2: Genes with scores of four in the respective cancer types. The absolute value was taken into account for the combined score.

| Cancer type | Combined score | OG score | TS score |
| --- | --- | --- | --- |
| BLCA |  |  |  |
| BRCA | MT3, BRCA2 |  | CTRB1, MT3, RB1, BRCA2 |
| COAD | SNTB1,AZGP1,  PUF60, STK31 | SNTB1, RB1, AZGP1, PUF60, STK31 |  |
| GBM |  |  | PTEN |
| HNSC |  |  |  |
| KIRC | NFATC4, CAMKV | TRRAP, FOXP2, CTNNA1 | NFATC4, TSHR, SLC25A29, BTBD6, BRF1, CAMKV |
| LUAD |  |  | RB1, SMARCA4 |
| LUSC |  |  |  |
| OV |  |  | RB1 |
| READ |  |  |  |
| UCEC |  |  |  |

Table S3: Wilcoxon test p-values for difference of tumor suppressor scores between frequently mutated genes identified by Wood *et al*. and all other human genes. The first column contains the mutation set based on which frequently mutated genes were called. The second column contains the cancer types used for scoring genes in our study.

|  |  | Tumor suppressor score | CNA | Meth | Mut | shRNA | Expr |
| --- | --- | --- | --- | --- | --- | --- | --- |
| Breast | all | 3.40*10^-10^ | 0.022 | 1.80*10^-09^ | 1.30*10^-11^ | 5.80*10^-03^ | 0.53 |
|  | BRCA | 9.10*10^-05^ | 0.33 | 8.70*10^-06^ | 2.60*10^-02^ | 4.00*10^-02^ | 0.0051 |
| Colon | all | 4.50*10^-23^ | 0.0026 | 1.10*10^-25^ | 6.60*10^-30^ | 5.40*10^-04^ | 0.00087 |
|  | COAD | 2.10*10^-09^ | 0.25 | 3.80*10^-02^ | 1.20*10^-32^ | 9.80*10^-01^ | 0.011 |
| Both | all | 4.30*10^-29^ | 0.00024 | 2.10*10^-30^ | 1.40*10^-35^ | 7.60*10^-06^ | 0.0098 |

|  |  | Oncogene  Score | CNA | Meth | Mut | shRNA | Expr |
| --- | --- | --- | --- | --- | --- | --- | --- |
| Breast | all | 9.90*10^-08^ | 0.16 | 1.40*10^-07^ | 7.40*10^-02^ | 1.40*10^-02^ | 0.013 |
|  | BRCA | 1.30*10^-06^ | 0.28 | 4.70*10^-06^ | 4.00*10^-03^ | 2.40*10^-01^ | 0.069 |
| Colon | all | 7.10*10^-12^ | 0.7 | 1.40*10^-18^ | 9.70*10^-03^ | 1.20*10^-03^ | 0.36 |
|  | COAD | 0.380 | 0.34 | 2.30*10^-02^ | 4.20*10^-10^ | 1.10*10^-01^ | 0.021 |
| Both | all | 3.70*10^-17^ | 0.52 | 1.70*10^-22^ | 1.20*10^-02^ | 4.30*10^-05^ | 0.29 |

Table S4: Wilcoxon test p-values for significance of difference of oncogene scores between frequently mutated genes identified by Wood *et al*. and all other human genes. The first column contains the mutation set based on which frequently mutated genes were called. The second column contains the cancer types used for scoring genes in our study.

Table S5: Wilcoxon test p-values for significance of difference of oncogene (OG) and tumor suppressor (TS) gene scores between the high confidence driver genes identified by Tamborero *et al*. and all other human genes.

|  | Score | CNA | Meth | Mut | shRNA | Expr |
| --- | --- | --- | --- | --- | --- | --- |
| OG | 1.1*10^-21^ | 0.031 | 6*10^-08^ | 2.4*10^-65^ | 7.9*10^-20^ | 1.4*10^-07^ |
| TS | 1.2*10^-24^ | 1.1*10^-08^ | 6.3*10^-06^ | 1.7*10^-224^ | 6.3*10^-19^ | 0.018 |

Table S6: Wilcoxon test p-values for significance of difference of tumor suppressor scores between frequently mutated genes identified by Lawrence *et al*. and all other human genes. The first row takes into account average scores across all cancer types for all identified genes. The remaining rows take into account scores for each cancer type for the respective genes.

|  | Tumor Suppressor Score | CNA | Meth | Mut | shRNA | Expr |
| --- | --- | --- | --- | --- | --- | --- |
| combined | 1.70*10^-12^ | 2.60*10^-05^ | 2.30*10^-02^ | 7.50*10^-203^ | 5.70*10^-08^ | 1.50*10^-01^ |
| BLCA | 1.80*10^-05^ | 2.00*10^-04^ | 0.075 | 8.10*10^-67^ | 5.70*10^-02^ | 0.47 |
| BRCA | 1.00*10^-05^ | 5.10*10^-04^ | 0.15 | 1.20*10^-133^ | 1.50*10^-01^ | 0.53 |
| COAD | 7.10*10^-05^ | 1.20*10^-03^ | 0.69 | 7.40*10^-39^ | 4.20*10^-05^ | 0.11 |
| UCEC | 9.40*10^-05^ | 7.10*10^-02^ | 0.033 | 1.40*10^-112^ | 1.50*10^-02^ | 0.29 |
| GBM | 8.40*10^-04^ | 3.30*10^-01^ | 0.044 | 1.30*10^-34^ | 2.60*10^-03^ | 0.36 |
| HNSC | 4.30*10^-05^ | 4.50*10^-05^ | 0.85 | 8.60*10^-109^ | 3.70*10^-02^ | 0.00057 |
| KIRC | 1.30*10^-02^ | 1.50*10^-02^ | 0.46 | 1.20*10^-43^ | 4.10*10^-01^ | 0.49 |
| OV | 3.00*10^-02^ | 7.70*10^-03^ | 0.34 | 4.80*10^-17^ | 1.20*10^-01^ | 0.14 |
| LUAD | 6.70*10^-04^ | 6.60*10^-03^ | 0.35 | 2.10*10^-56^ | 1.30*10^-01^ | 0.4 |
| LUSC | 1.00*10^-05^ | 4.50*10^-05^ | 0.054 | 2.80*10^-78^ | 1.60*10^-01^ | 0.47 |

Table S7: Wilcoxon test p-values for significance of difference of oncogene scores between frequently mutated genes identified by Lawrence *et al*. and all other human genes. The first row takes into account average scores across all cancer types for all identified genes. The remaining rows take into account scores for each cancer type for the respective genes.

|  | Oncogene  Score | CNA | Meth | Mut | shRNA | Expr |
| --- | --- | --- | --- | --- | --- | --- |
| combined | 6.30*10^-16^ | 1.30*10^-02^ | 1.80*10^-06^ | 1.30*10^-36^ | 2.80*10^-09^ | 1.90*10^-04^ |
| BLCA | 2.00*10^-02^ | 0.42 | 0.012 | 2.10*10^-23^ | 5.50*10^-02^ | 0.98 |
| BRCA | 6.90*10^-03^ | 0.52 | 0.029 | 4.00*10^-29^ | 4.40*10^-02^ | 0.22 |
| COAD | 1.60*10^-04^ | 0.82 | 0.35 | 5.90*10^-51^ | 7.50*10^-06^ | 0.3 |
| UCEC | 2.00*10^-06^ | 0.032 | 0.0041 | 4.60*10^-35^ | 2.60*10^-03^ | 0.089 |
| GBM | 2.10*10^-05^ | 0.29 | 0.0065 | 1.30*10^-21^ | 3.50*10^-04^ | 0.017 |
| HNSC | 3.60*10^-03^ | 0.66 | 0.39 | 1.10*10^-10^ | 5.60*10^-03^ | 0.0025 |
| KIRC | 9.50*10^-03^ | 0.69 | 0.023 | 3.70*10^-10^ | 2.00*10^-01^ | 0.36 |
| OV | 1.40*10^-03^ | 0.088 | 0.18 | 4.50*10^-21^ | 5.90*10^-02^ | 0.085 |
| LUAD | 2.30*10^-04^ | 0.0079 | 0.11 | 6.40*10^-56^ | 4.50*10^-02^ | 0.15 |
| LUSC | 2.80*10^-02^ | 0.49 | 0.063 | 2.70*10^-15^ | 4.00*10^-02^ | 0.48 |

Table S8: Keywords used for assigning cell lines to TCGA cancer types. The columns contain the TCGA cancer type abbreviation, the CCLE tumor site annotation and the CCLE histological subtype annotation in this order.

| Cancer Type | Site.Primary | Hist.Subtype1 |
| --- | --- | --- |
| BLCA | urinary_tract | transitional_cell_carcinoma |
| BRCA | Breast | --- |
| COAD | large_intestine | --- |
| GBM | central_nervous_system | astrocytoma, astrocytoma_Grade_III, astrocytoma_Grade_IV, gliosarcoma, oligodendroglioma |
| HNSC | upper_aerodigestive_tract | squamous_cell_carcinoma |
| KIRC | kidney | clear_cell_renal_cell_carcinoma |
| LUAD | Lung | adenocarcinoma bronchioloalveolar_adenocarcinoma |
| LUSC | Lung | squamous_cell_carcinoma mixed_adenosquamous_carcinoma |
| OV | ovary | --- |
| READ | rectal | --- |
| UCEC | endometrium | --- |


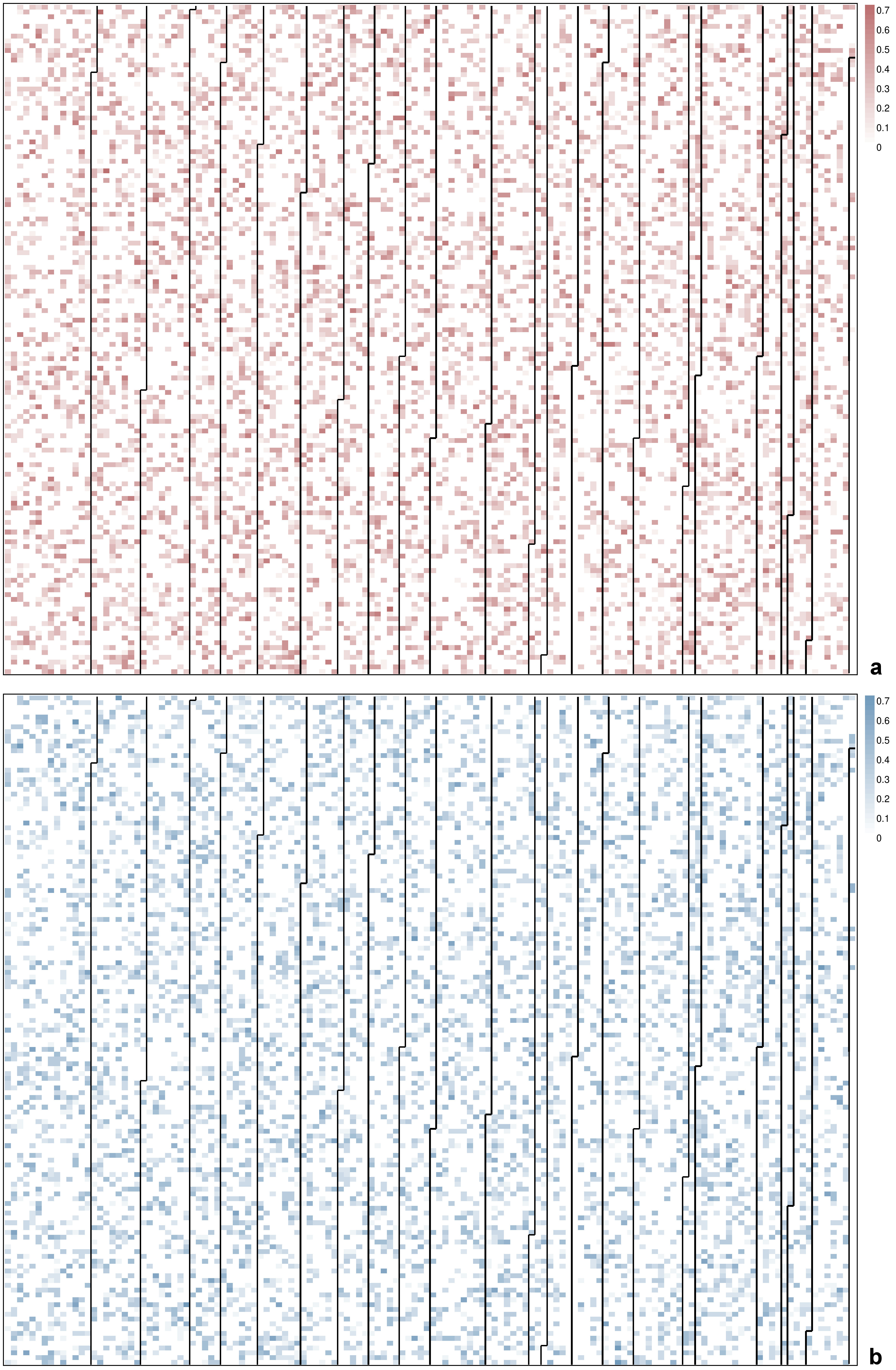
Figure S1: Heatmap of oncogene (a) and tumor suppressor (b) score based on shRNA knockdown data averaged across all 11 cancer types. Genes are plotted column-wise sorted according to chromosomal location, starting with the telomere of chromosome 1p in the upper left corner. Lines delineate the border between chromosomes.


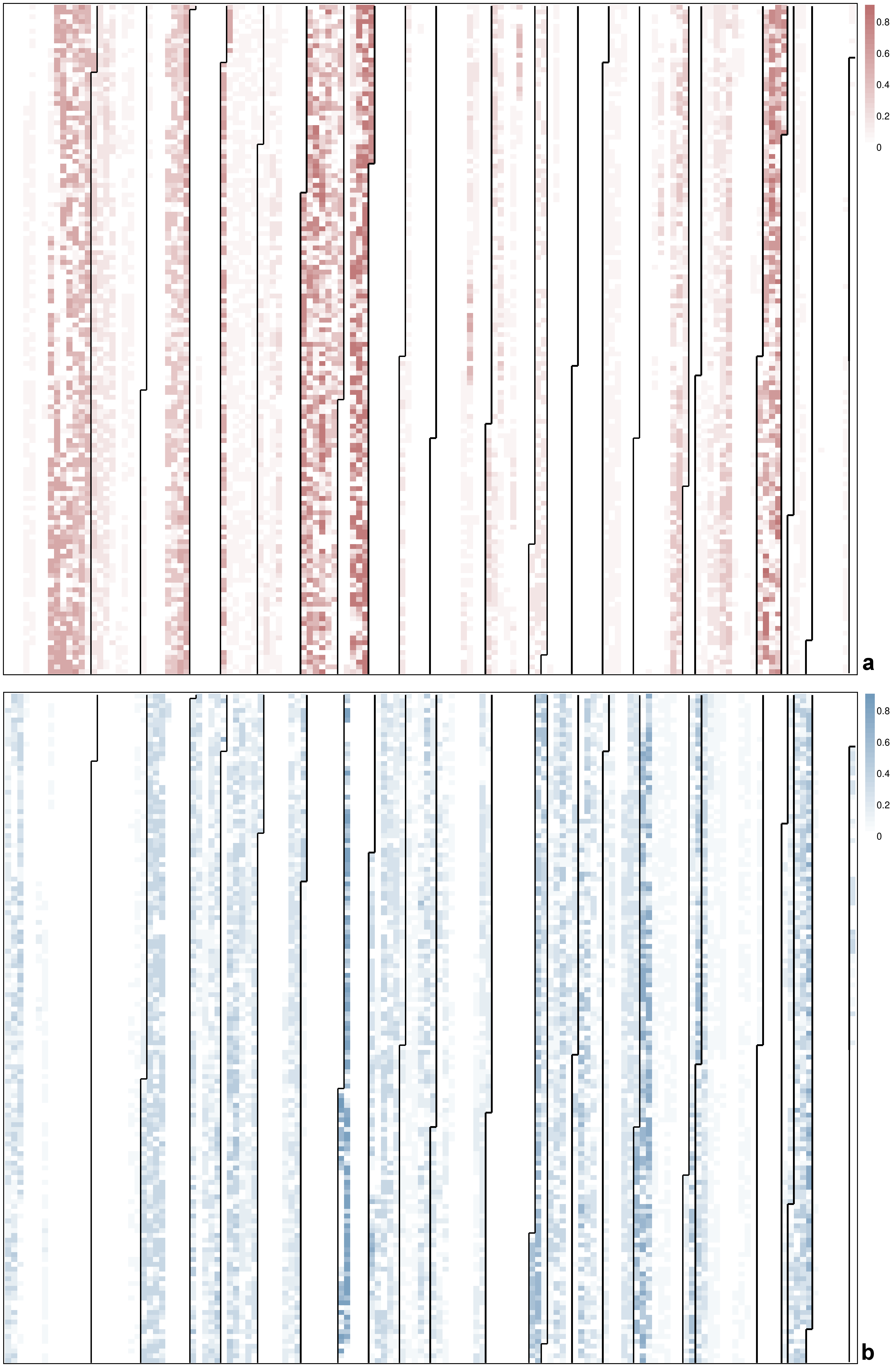
Figure S2: Heatmap of oncogene (a) and tumor suppressor (b) score based on DNA copy number data averaged across all 11 cancer types. Genes are plotted column-wise sorted according to chromosomal location, starting with the telomere of chromosome 1p in the upper left corner. Lines delineate the border between chromosomes.


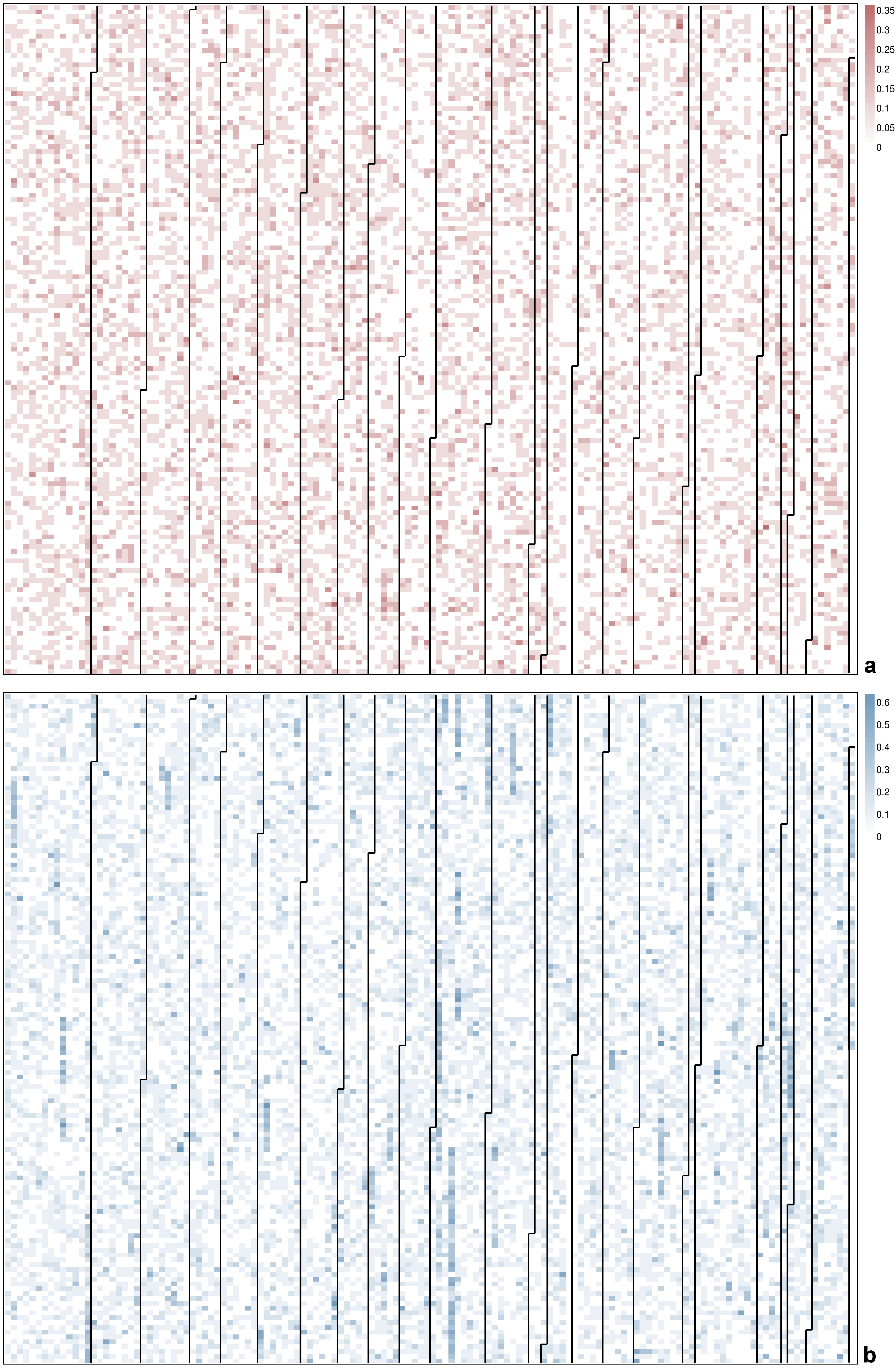
Figure S3: Heatmap of oncogene (a) and tumor suppressor (b) score based on gene expression data averaged across all 11 cancer types. Genes are plotted column-wise sorted according to chromosomal location, starting with the telomere of chromosome 1p in the upper left corner. Lines delineate the border between chromosomes.


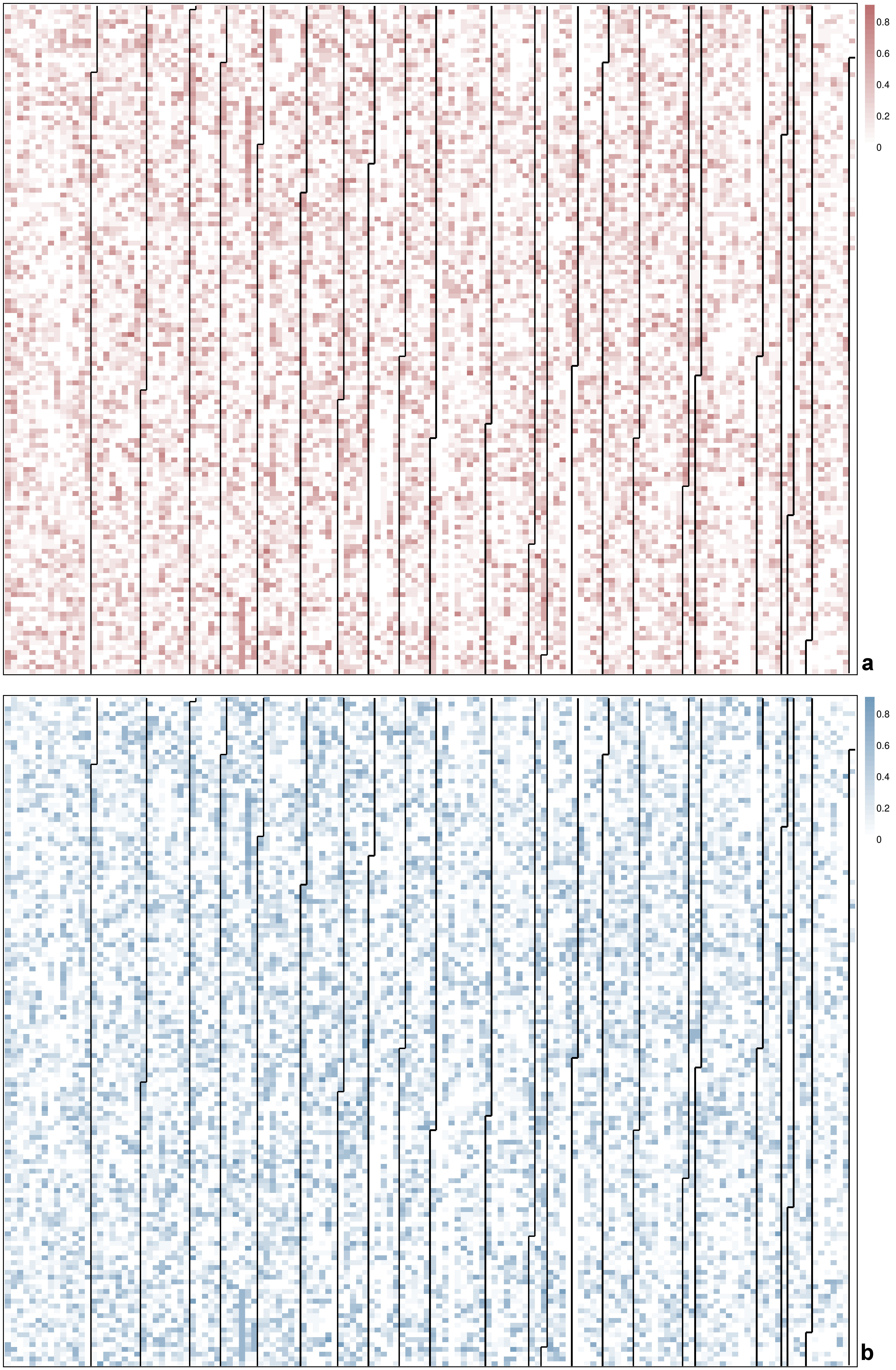
Figure S4: Heatmap of oncogene (a) and tumor suppressor (b) score based on DNA methylation data averaged across all 11 cancer types. Genes are plotted column-wise sorted according to chromosomal location, starting with the telomere of chromosome 1p in the upper left corner. Lines delineate the border between chromosomes.


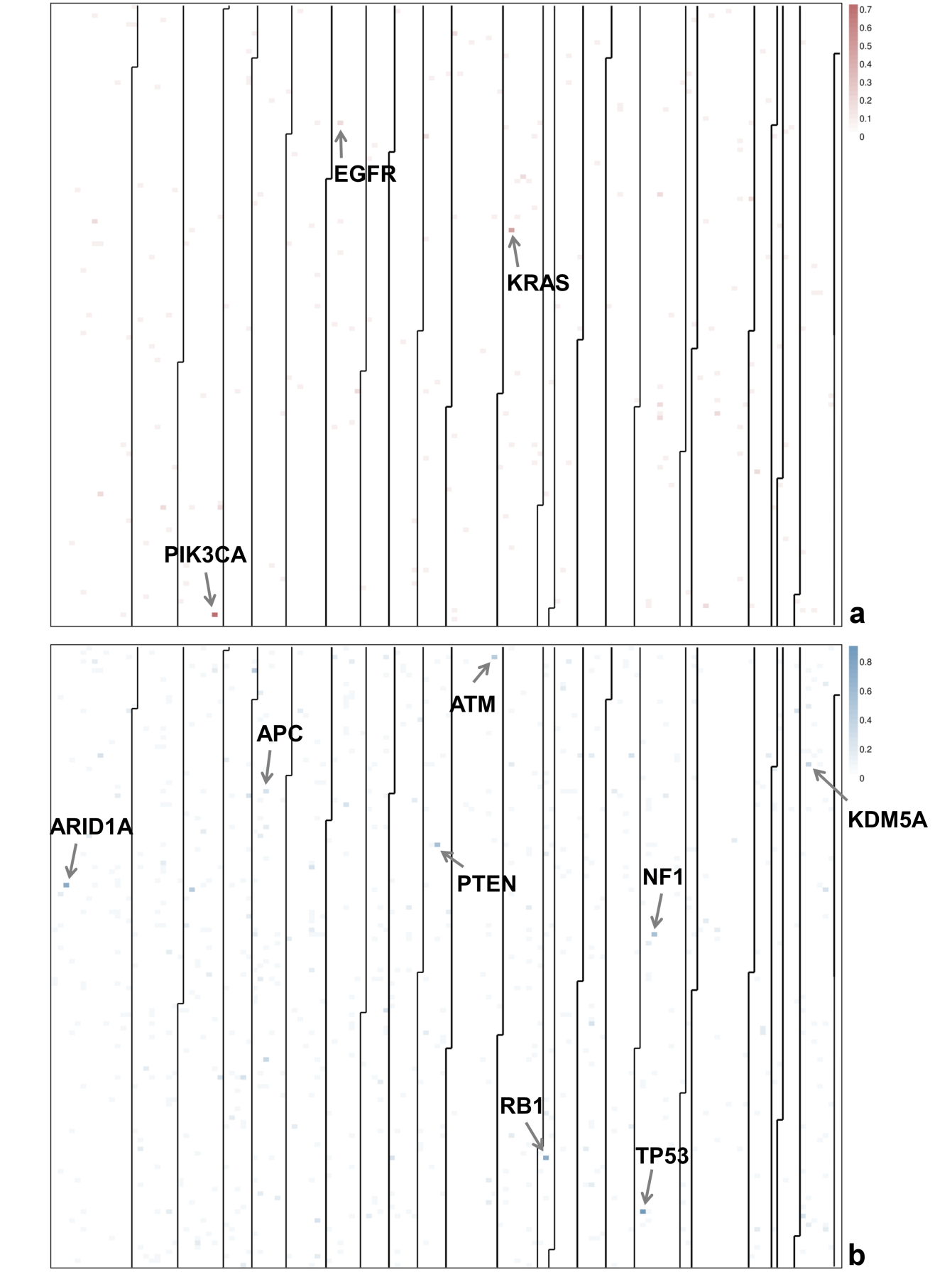
Figure S5: Heatmap of oncogene (a) and tumor suppressor (b) score based on somatic mutation data averaged across all 11 cancer types. Genes are plotted column-wise sorted according to chromosomal location, starting with the telomere of chromosome 1p in the upper left corner. Lines delineate the border between chromosomes. Some well-known cancer genes are highlighted.

Figure S6: Aberration profiles of STK31 and MSRA across all cancer types. Colored boxes indicate that the given gene was aberrated for the respective data type in the associated cancer. Marginal histograms indicate the number of different cancer types with aberrations in the given gene.
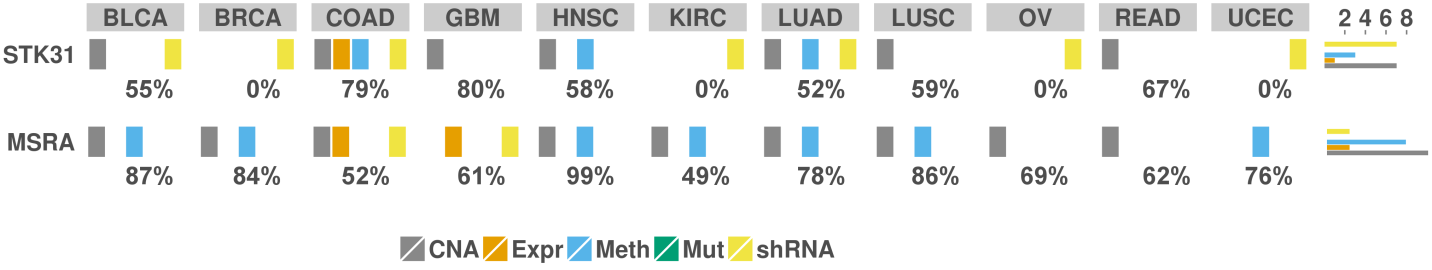
 The percentage of tumor samples which is estimated to harbor aberrations in the two genes is indicated for each cancer type.


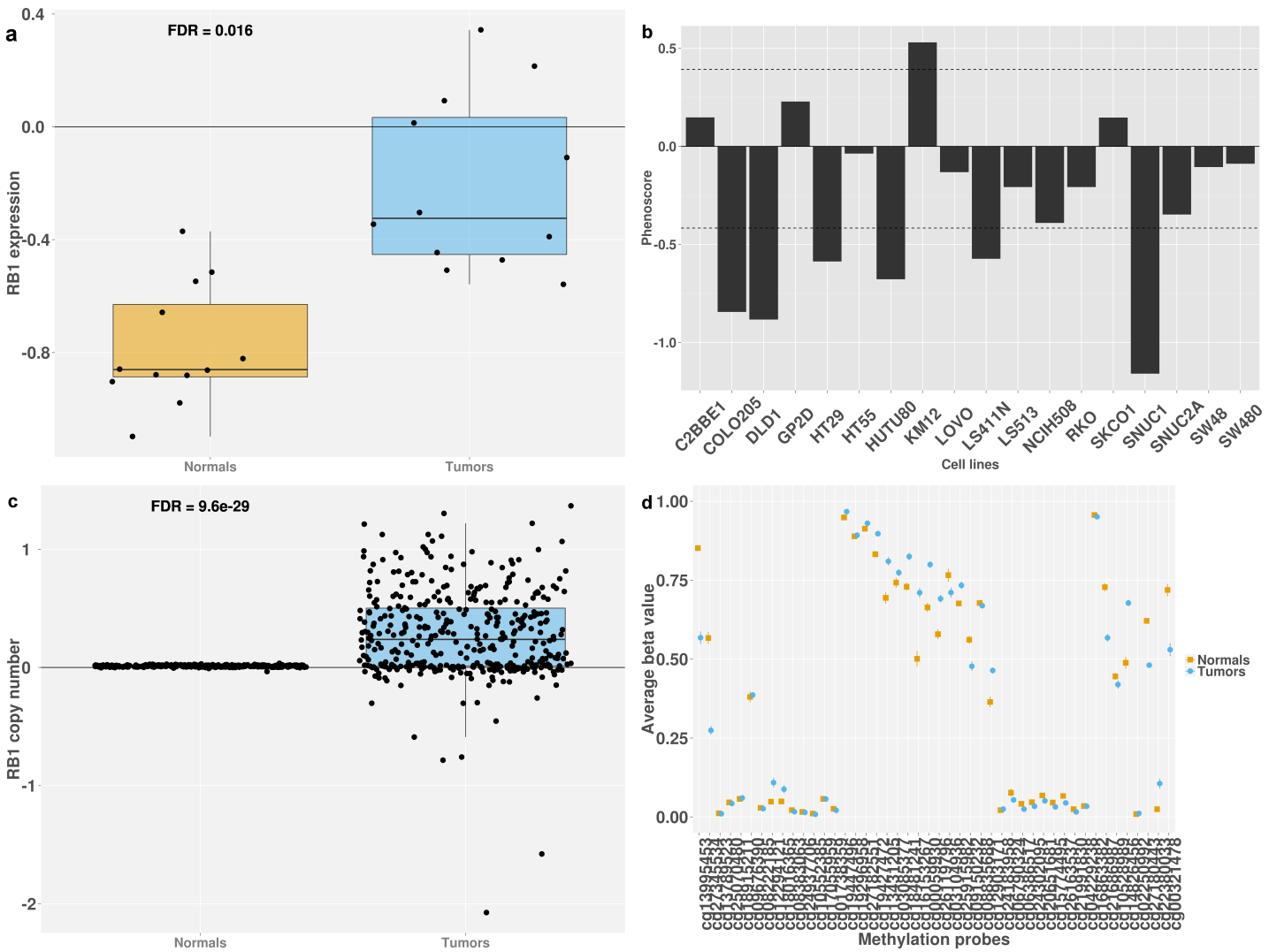


Figure S7: Data for *RB1* prioritization in COAD samples. (a) Boxplot of expression of tumor samples (blue) and matched normal (orange) COAD samples. (b) Barplot showing growth differences of colon cell lines as measured in Project Achilles after knock-down of *RB1*. (c) Boxplot of copy number values in TCGA COAD samples. (d) Methylation beta values at all probes across the *RB1* gene in TCGA COAD tumors (blue) and normal samples (orange). Beta value range from 0 (unmethylated) to 1 (fully methylated).


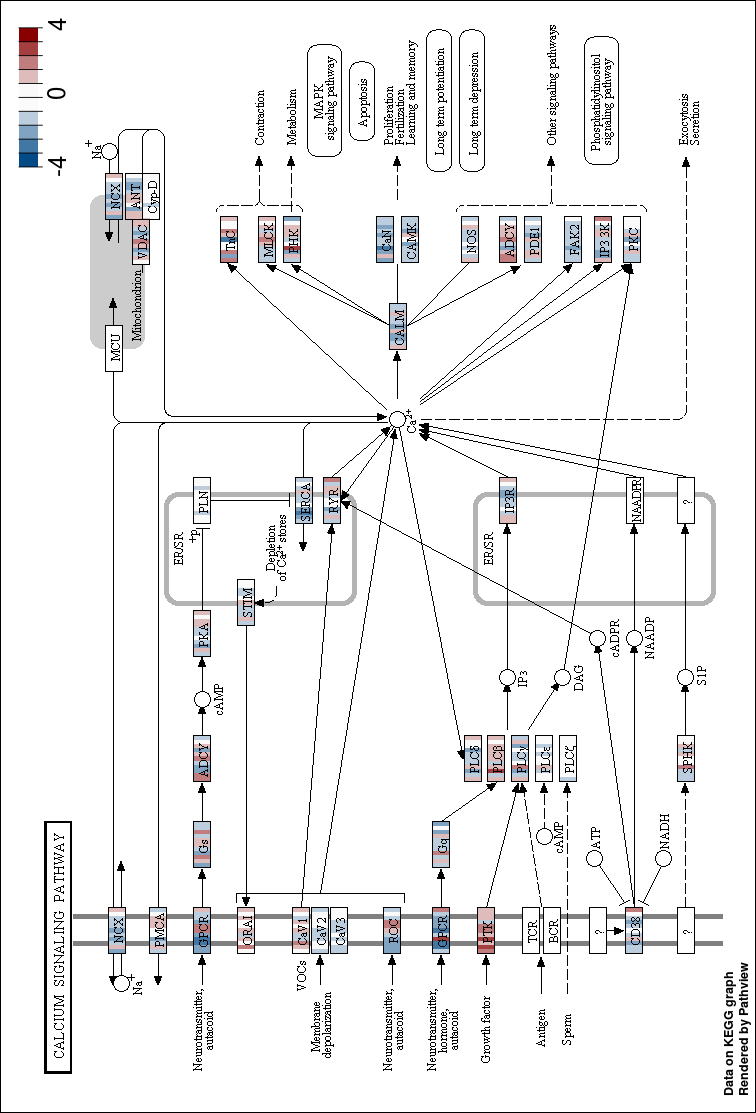


Figure S8: Map of the KEGG calcium signaling pathway with combined scores for individual genes. Negative scores (blue) indicate higher tumor suppressor scores and positive score (red) higher oncogene scores. Each gene node is divided into 11 fields showing scores for each cancer in the following order: BLCA, BRCA, COAD, GBM, HNSC, KIRC, LUAD, LUSC, OV, READ, UCEC. If several genes mapped to a node, the maximum absolute value was selected.


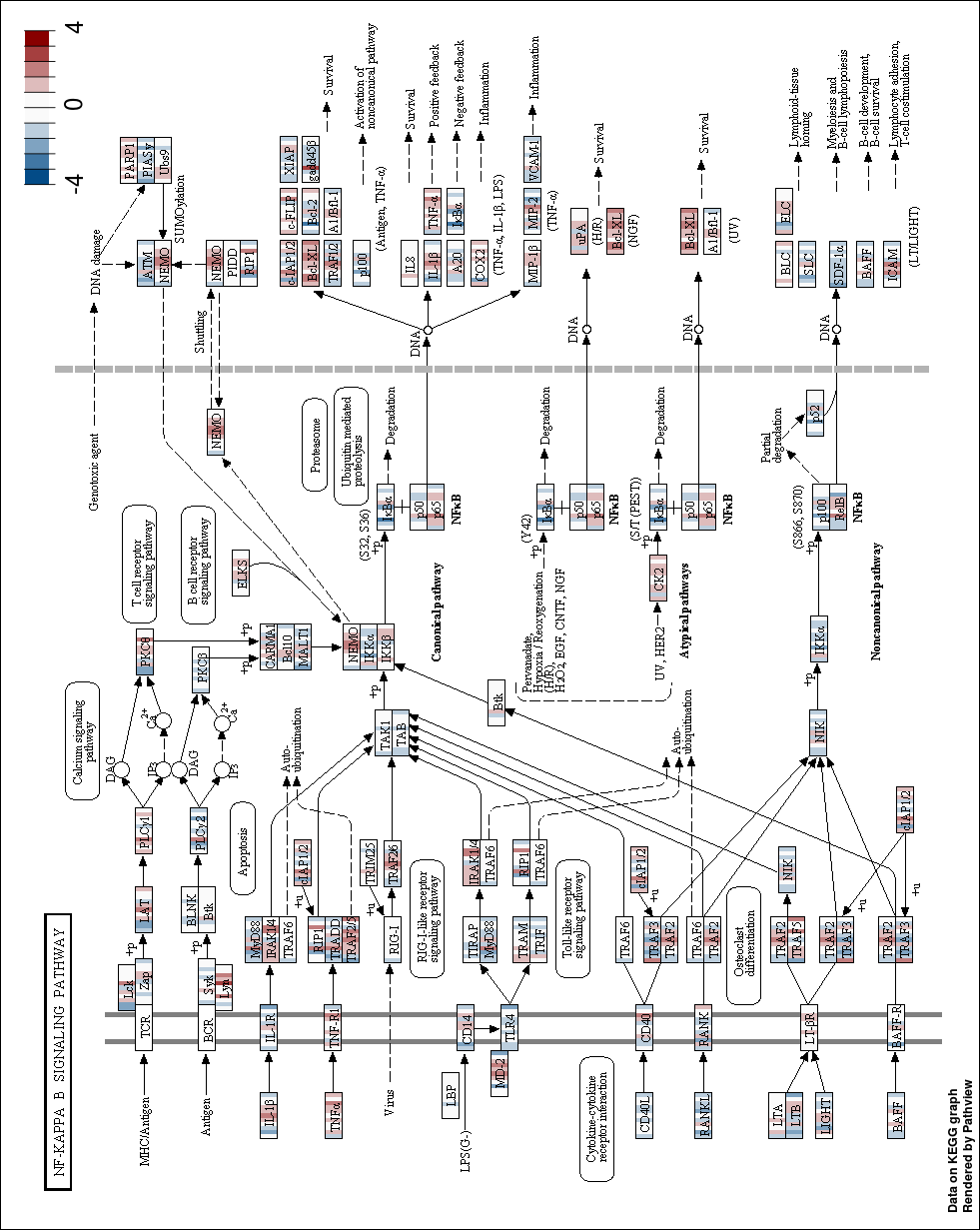
Figure S9: Map of the KEGG NF-Kappa B signaling pathway with combined scores for individual genes. Negative scores (blue) indicate higher tumor suppressor scores and positive score (red) higher oncogene scores. Each gene box is divided into 11 fields showing the scores for each cancer in the following order: BLCA, BRCA, COAD, GBM, HNSC, KIRC, LUAD, LUSC, OV, READ, UCEC. If several genes mapped to a node, the maximum absolute value was selected.


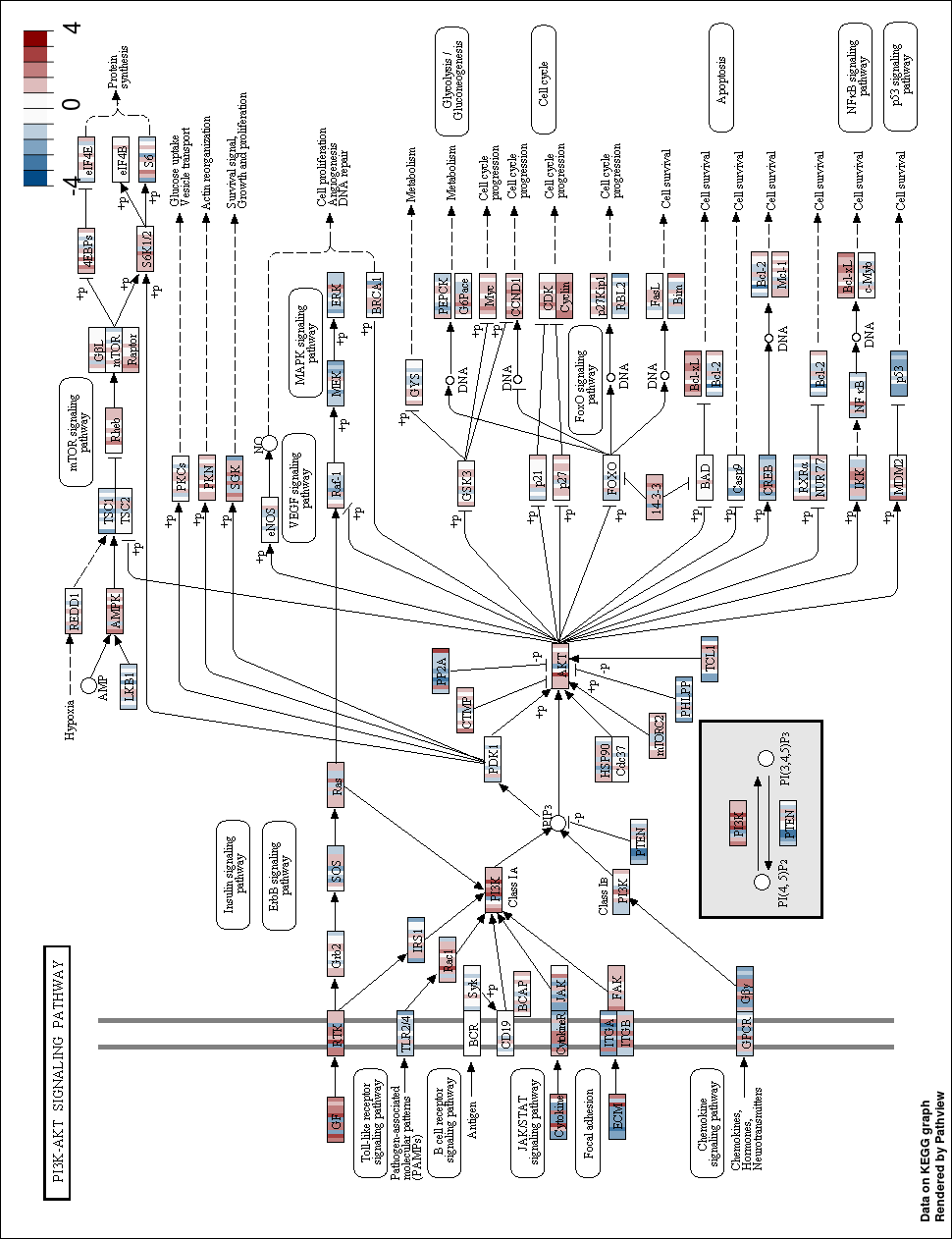


Figure S10: Map of the KEGG PI3K-AKT signaling pathway with combined scores for individual genes. Negative scores (blue) indicate higher tumor suppressor scores and positive score (red) higher oncogene scores. Each gene box is divided into 11 fields showing the scores for each cancer in the following order: BLCA, BRCA, COAD, GBM, HNSC, KIRC, LUAD, LUSC, OV, READ, UCEC. If several genes mapped to a node, the maximum absolute value was selected.


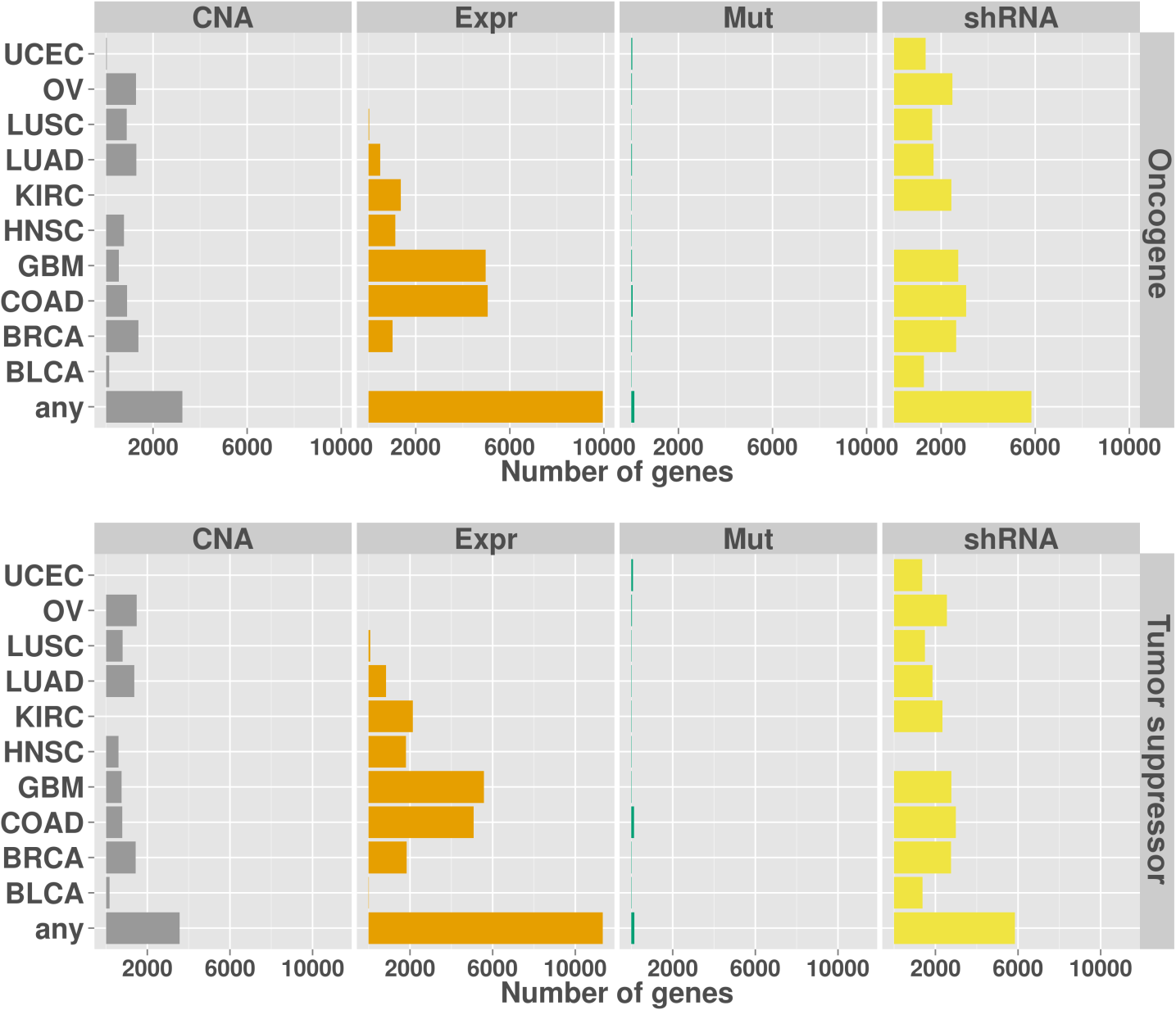
Figure S11: Number of genes with oncogene-like and tumor suppressor-like alterations in cancer cell lines for the different data types across all cancer types. Data types are depicted in panels and cancer types in rows. The row labeled “any” indicates the number of genes with a aberration in any cancer type.


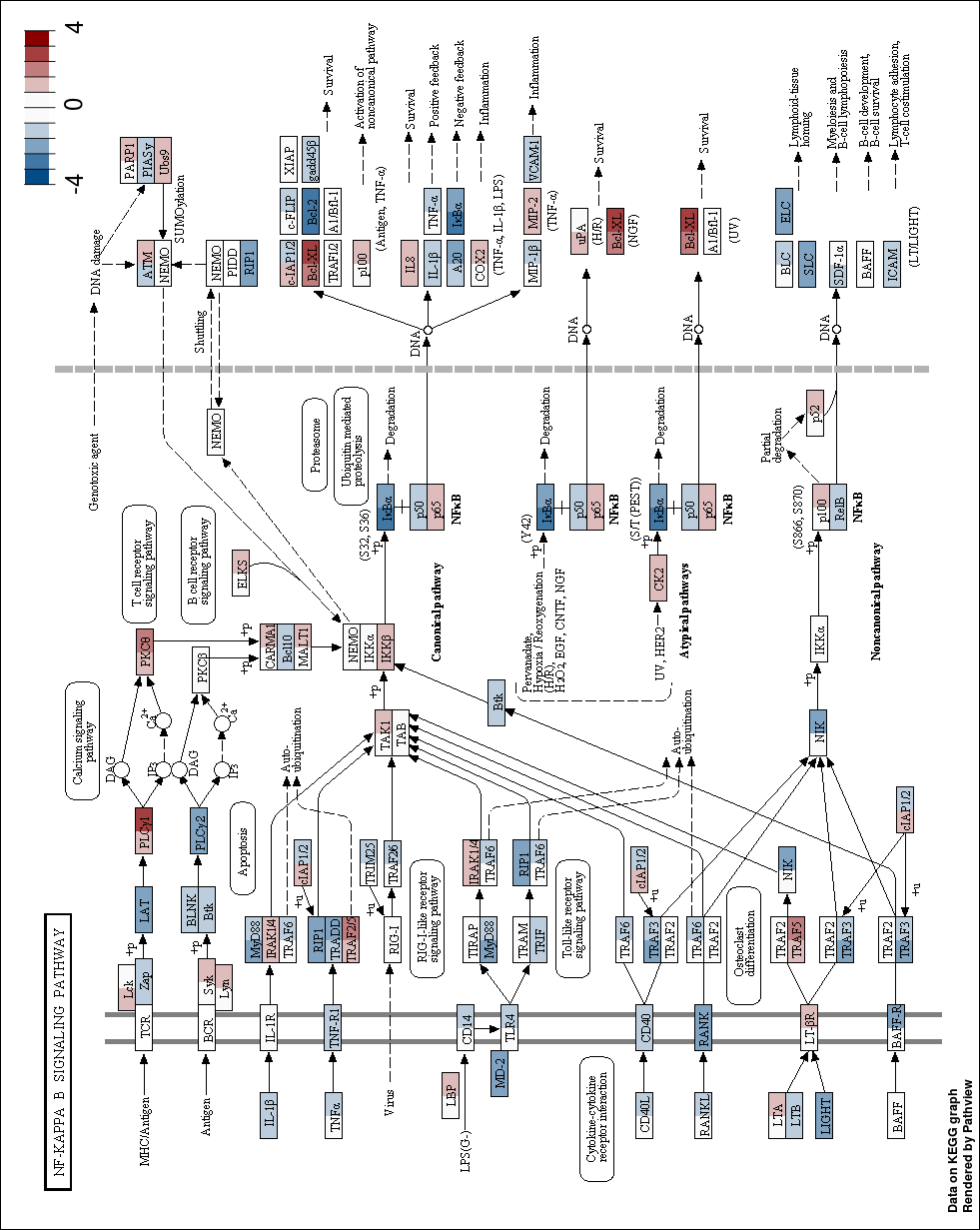


Figure S12: Map of the KEGG NF-Kappa B signaling pathway with combined scores for aberrations in COAD. Each gene box is divided into 2 fields showing the scores for TCGA tumor samples (left) and CCLE cell lines (right). Negative scores (blue) indicate higher tumor suppressor scores and positive score (red) higher oncogene scores. If several genes mapped to a node, the maximum absolute value was selected.


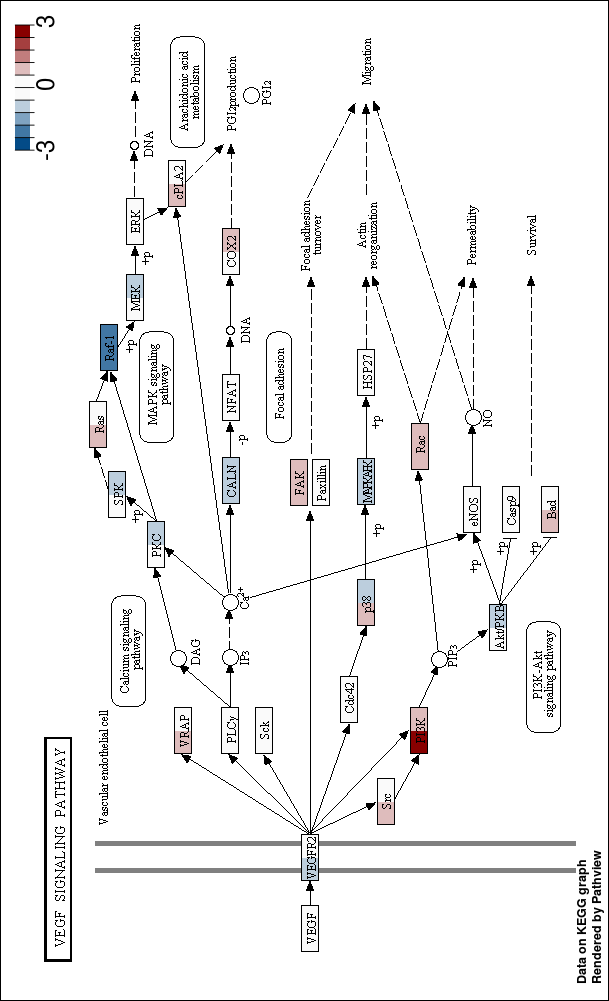


Figure S13: Map of the KEGG VEGF signaling pathway with combined scores for aberrations in HNSC. Each gene box is divided into 2 fields showing the scores for TCGA tumor samples (left) and CCLE cell lines (right). Negative scores (blue) indicate higher tumor suppressor scores and positive score (red) higher oncogene scores. If several genes mapped to a node, the maximum absolute value was selected.


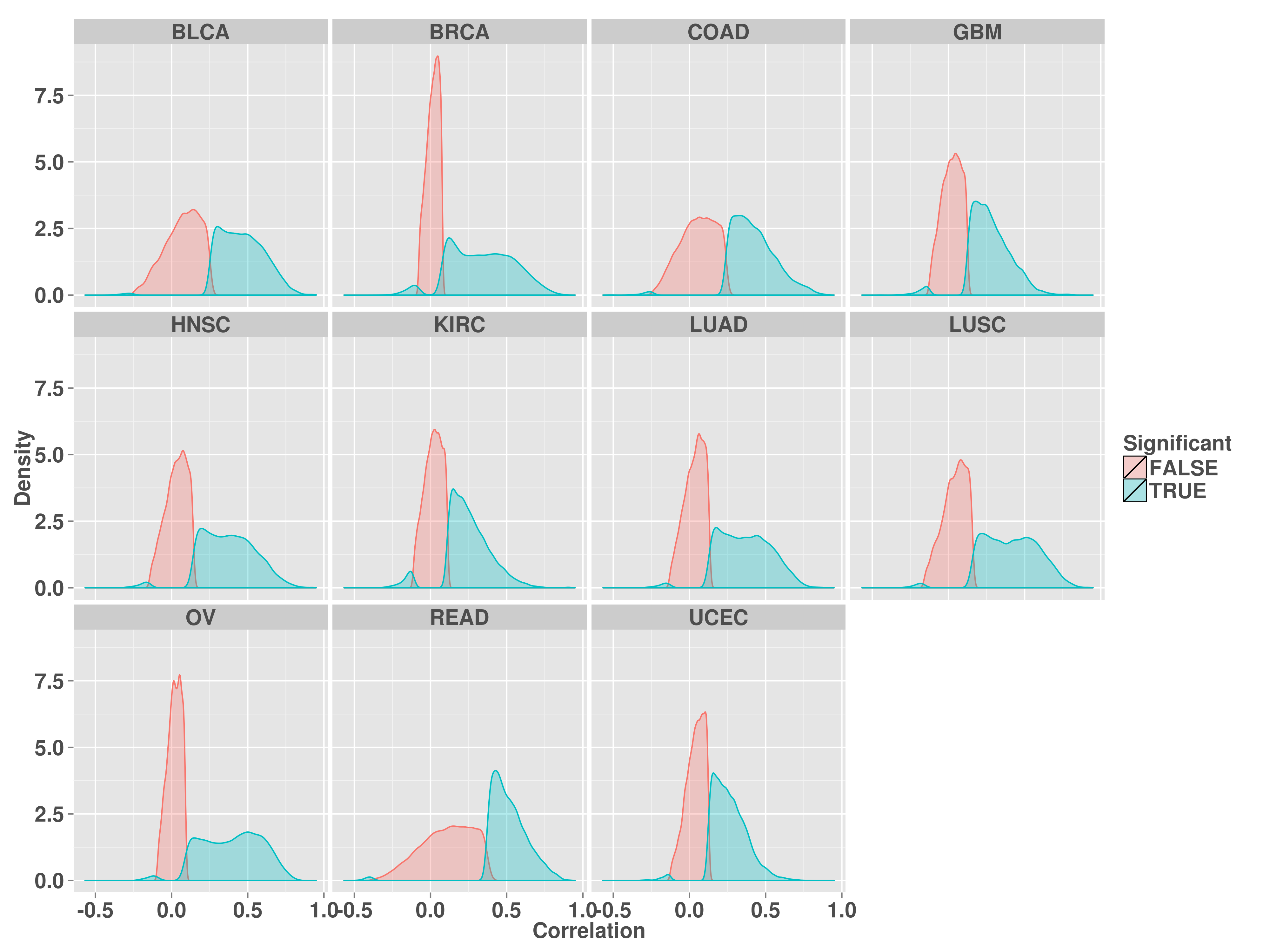


Figure S14: Density plots of correlation values between DNA copy number and expression for each gene split by cancer type. Correlations were divided into significant (FDR < 0.05, blue) and not significant (FDR >= 0.05, red).


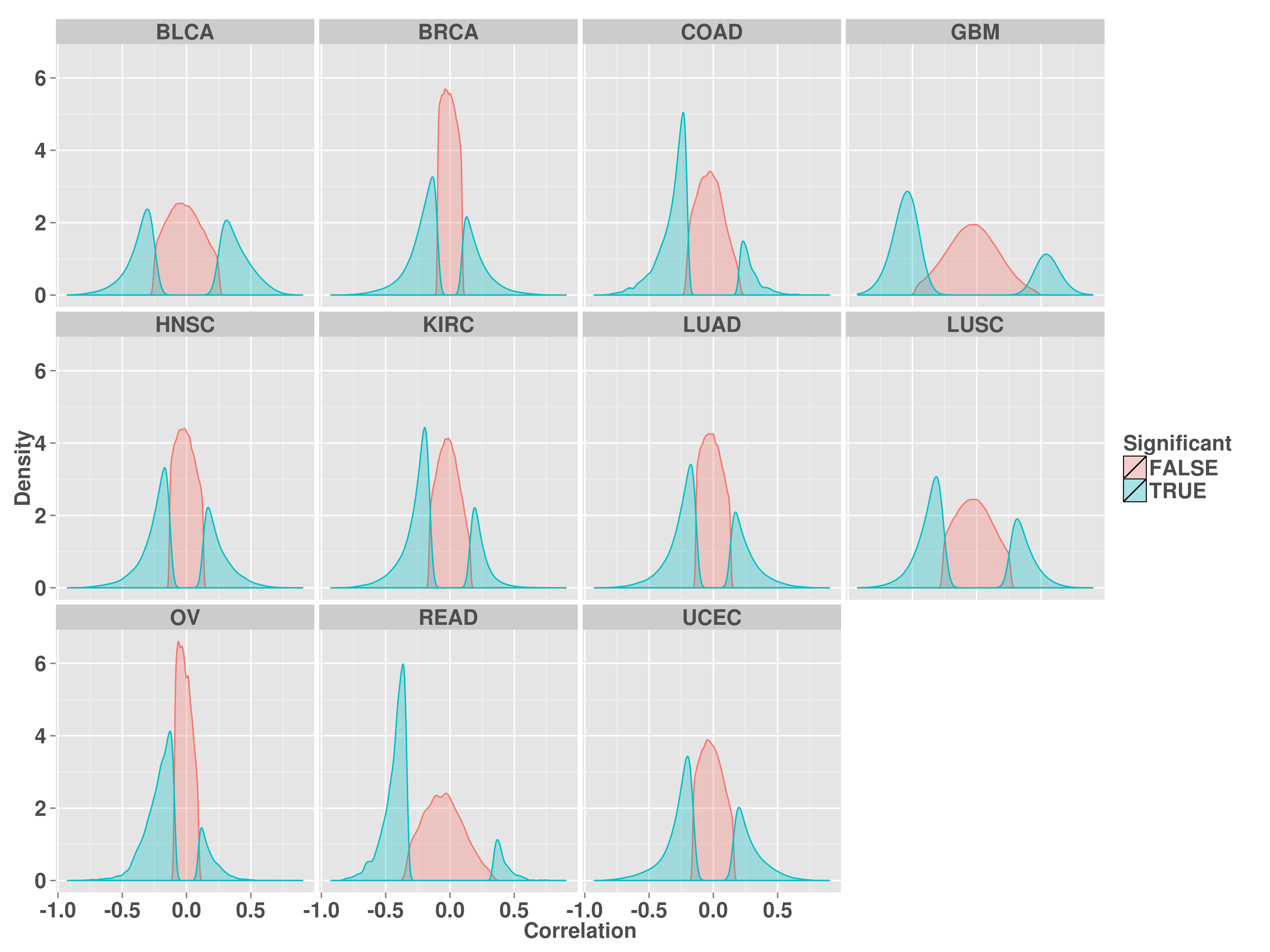
Figure S15: Density plots of correlation values between DNA methylation and expression for each methylation probe split by cancer type. Correlations were divided into significant (FDR < 0.05, blue) and not significant (FDR >= 0.05, red).
